# Association of the *miR-146a*, *miR-149*, *miR-196a2* and *miR-499* polymorphisms with susceptibility to pulmonary tuberculosis in the Chinese Uygur, Kazak and Southern Han populations

**DOI:** 10.1186/s12879-015-0771-9

**Published:** 2015-02-05

**Authors:** Xing Zhang, Yanyuan Li, Xiang Li, Wanjiang Zhang, Zhifen Pan, Fang Wu, Chong Wang, Zhongliang Chen, Tingting Jiang, Dandan Xu, Zepeng Ping, Jiyan Liu, Changming Liu, Zhongjie Li, Ji-Cheng Li

**Affiliations:** Institute of Cell Biology, Zhejiang University, No. 866, Yuhangtang Road, Hangzhou, 310058 China; Department of Pathology, First Affiliated Hospital, Zhejiang University, Hangzhou, 310003 China; Key Laboratory of Gastroenteropathy, Zhejiang Province People’s Hospital, Hangzhou, 310012 China; Department of Pathophysiology, Shihezi University School of Medicine, Xinjiang, 832000 China; Department of Tuberculosis, Jiaxing First Hospital, Jiaxing, 314000 China

**Keywords:** MicroRNAs, Pulmonary tuberculosis, Single-nucleotide polymorphism, Kazak, Uygur, Southern Han

## Abstract

**Background:**

Single nucleotide polymorphisms (SNPs) within precursor microRNAs (miRNAs) can affect miRNAs expression, and may be involved in the pathogenesis of pulmonary tuberculosis (TB). This study aimed to investigate potential associations between the four precursor *miRNA* SNPs (*miR-146a* C > G, *miR-149* T > C, *miR-196a2* T > C, and *miR-499* T > C) and susceptibility to pulmonary TB in the Chinese Uygur, Kazak, and Southern Han populations.

**Methods:**

A case-control study was performed on Chinese Uygur (n = 662), Kazak (n = 612), and Southern Han (n = 654) populations using the PCR-PFLR method. The allele and genotype frequencies for all populations were analyzed. Linkage disequilibrium was performed, and different models of inheritance were tested.

**Results:**

The allele and genotype frequencies of the *miR-499* SNP were significantly different between the TB patients group and the healthy control group in the Uygur population, and were found to be codominant, dominant, recessive and additive models in association with pulmonary TB. The haplotype CTCC showed significant correlation with pulmonary TB. The allele and genotype frequencies of *miR-146a* and *miR-196a2* SNPs were significantly different between the two groups in the Kazak population. The *miR-146a* SNP was found to be codominant, recessive and additive models, whereas, the *miR-196a2* SNP was found to be codominant, dominant, and additive models in association with pulmonary TB. The haplotypes TCCC and CCCT showed significant correlation with pulmonary TB.

**Conclusions:**

The results suggested that susceptibility to pulmonary TB may be closely related to individual differences caused by genetic factors among different ethnic groups in China.

**Electronic supplementary material:**

The online version of this article (doi:10.1186/s12879-015-0771-9) contains supplementary material, which is available to authorized users.

## Background

Pulmonary tuberculosis (TB) caused by *Mycobacterium tuberculosis* (*Mtb*) is a chronic disease that can present serious threats to human health. According to the World Health Organization (WHO) global TB report, an estimated 8.56 million of people developed active form of TB in 2012 and 1.26 million died from the disease (including 320 000 deaths among HIV-positive people). Therefore, TB is still a leading cause of death in humans [1]. During the same year, an estimated 1.37 million cases of TB occurred in China, accounting for 18% of all TB infections worldwide. About 130 000 people die each year from TB in China, which has the world’s second largest TB epidemic, only behind India [1]. The Uygur and Kazak populations in the Xinjiang Uygur Autonomous Region of northwest China are seriously affected by TB. The prevalence rate of active, sputum smear-positive, and sputum culture-positive pulmonary TB in these populations have been found to be 464/10000, 160/10000, and 38/10000, respectively, and were 1.26, 1.51 and 3.88 times higher than the national average, respectively. The prevalence rate of active, sputum smear-positive and sputum culture-positive pulmonary TB in the Chinese Uygur and Kazak populations has also been found to be 12.4%, 16.9% and 18.4% higher than the Chinese Han population, respectively. Therefore, molecular epidemiological studies on pulmonary TB in the Xinjiang region will contribute to accurately identify the outbreak and spread of disease in China [2].

It is estimated that about one-third of the global population is infected with *Mtb*, of whom 5-15% will develop active pulmonary TB [3]. This suggests that individual differences caused by genetic factors may play an important role in susceptibility to TB. Understanding the effect of individual factors on the immune mechanisms of the pathogen will help to prevent and treat TB. In recent years, single nucleotide polymorphisms (SNPs) in genes associated with susceptibility to TB such as Toll-like receptor (*TLR2*, *TLR4* and *TLR8*) [4,5], NOD-like receptor (*NOD2*) [6], mannose receptor (*MRC1*) [7,8], and cytotoxic T lymphocyte antigen-4 (*CTLA4*) [9] have been reported. However, to date, miRNA SNPs conferring susceptibility to pulmonary TB have not yet been reported in the Chinese Uygur, Kazak, and Southern Han populations.

MicroRNAs (miRNAs) are a class of endogenous, small, non-coding RNAs of approximately 20~25 nucleotides, and have important gene-regulatory functions. Following transcription, mature miRNAs are generated through a series of coordinated processing events mediated by large protein complexes. Recent studies have shown that miRNAs are key regulators of gene expression [10]. Although only a few hundred miRNAs have been discovered, each miRNA could potentially regulate hundreds of target genes. It has been suggested that one-third of the human genes may be regulated by miRNAs [11]. The expression levels of miRNAs have also been shown to be associated with cancer [12], and other diseases [13]. In addition, miRNAs have been used as potential biomarkers for both non-communicable and communicable diseases [14,15]. The altered gene expression profiles in natural killer (NK) cells and macrophages from TB-infected patients and healthy controls have also been demonstrated to be regulated by miRNAs [16].

SNPs in precursor miRNAs (pre-miRNAs) can alter miRNAs processing and expression, and may contribute to the development and progression of disease. Recently, four potentially functional SNPs in pre-miRNAs (*miR-146a* C > G rs2910164, *miR-149* T > C rs2292832, *miR-196a2* T > C rs11614913, and *miR-499* T > C rs3746444) have been listed in the public miRNA database. MiRNA SNPs have been shown to be associated with susceptibility to a variety of human diseases [17-19]. Furthermore, the expression of miRNAs has also been found to be correlated with pulmonary TB [20-22]. Therefore, we speculated that a correlation exists between the miRNA SNPs and susceptibility to pulmonary TB.

The present study is the first to report that *miR-146a*, *miR-149*, *miR-196a2,* and *miR-499* SNPs can be associated with susceptibility to pulmonary TB in the Chinese Uygur, Kazak, and Southern Han populations. The study provides a new idea to clarify susceptibility to pulmonary TB among different ethnic groups in China, and may play an important role in controlling and preventing pulmonary TB.

## Methods

### Patients and control subjects

Patients with pulmonary TB were diagnosed according to the criteria described by the Ministry of Health, China [23]. A total of 301 Chinese Uygur pulmonary TB patients (157 males and 144 females; aged 18-70 years, mean age 40.1 ± 14.7 years) were recruited from the Second People’s Hospital of Aksu, Xinjiang and Wensu County People’s Hospital (China) between January 2009 and June 2012. For the Chinese Kazak population, a total of 251 pulmonary TB patients (154 males and 97 female; aged 20-70 years, mean age 37.1 ± 10.9 years) were recruited from the Tacheng District Hospital (Xinjiang, China) between December 2008 and June 2012. A total of 354 Chinese Southern Han pulmonary TB patients (aged 18-66 years, mean age 43.5 ± 12.0 years) were recruited from the Sixth Hospital of Shaoxing and Hangzhou Red Cross Hospital (Southern China) between December 2009 and June 2013.

All healthy controls were recruited from the same ethnic (Uygur, Kazak, and Han) populations and areas where patients were recruited. A total of 361 Uygur (219 males and 142 females; aged 20-65 years, mean age 37.0 ± 12.3 years) and 362 Kazak healthy control individuals (213 males and 149 females; aged 20-72 years, mean age 36.5 ± 9.6 years) were living in the Xinjiang autonomous region (China). A total of 300 Southern Han healthy control individuals (aged 19-64 years, mean age 46.1 ± 14.1 years) were recruited from the Zhejiang Hospital (Southern China) between October 2009 and July 2012 (Table 1).

**Table 1:** **Characteristics of Uygur, Kazak and Southern Han populations for** ***miR-146a*** **C > G,** ***miR-149*** **T > C,** ***miR-196a2*** **T > C and** ***miR-499*** **T > C SNPs detection**

**Characteristics**	**Uygur (n = 662)**	**Kazak (n = 613)**	**Southern Han (n = 654)**
**Pulmonary TB patients**	**N = 301**	**N = 251**	**N = 354**
Age, years range (mean ± SD)	18-70	20-70	18-65
	(40.1 ± 14.7)	(37.5 ± 10.9)	(43.5 ± 12.0)
Gender (female: male)	144: 157	97:154	165:189
Body mass index (mean ± SD)	20.7 ± 3.4	22.7 ± 3.9	26.3 ± 2.7
Sputum culture-proven	148(49.2%)	150(59.8%)	169(47.9%)
Chest X-ray and CT-proven	156(51.8%)	141(56.2%)	176(49.6%)
Pulmonary TB	301(100%)	251(100%)	354(100%)
Tuberculin skin test (>10 mm), no. (%)	216(71.8%)	199(79.3%)	268(75.7%)
TB history of relatives, no. (%)	31(10.5%)	36(14.6%)	63(17.7%)
BCG vaccination, no. (%)	166(55.2%)	125(49.8%)	190(53.8%)
**Controls**	**N = 361**	**N = 362**	**N = 300**
Age, years range (mean ± SD)	20-65	20-72	19-64
	(37.0 ± 12.3)	(36.5 ± 9.6)	(46.1 ± 14.1)
Gender (female: male)	142: 219	149: 213	137:163
Body mass index (mean ± SD)	23.0 ± 3.7	23.0 ± 3.9	24.6 ± 3.7
TB history of relatives, no. (%)	23(6.4%)	27(7.6%)	17(5.7%)
BCG vaccination, no. (%)	164(45.3%)	204(56.5%)	162(53.9%)

TB: tuberculosis; N: number of subjects; Data are reported as number with percentage in parentheses, unless otherwise stated.

The early morning fasting blood samples from all participants were collected in 5.0 ml EDTA tubes, and stored at -80°C. For each SNP, it was ensured that the pulmonary TB patients were similar to healthy controls in the ratio of gender, age, history of exposure to TB, vaccination, and purified protein derivative (PPD) skin test. The study was approved by the Ethics Committee of the Faculty of Medicine (Zhejiang University, China), and informed consents were obtained from all subjects before blood sampling.

### Genetic analyses

Genomic DNA was extracted from peripheral blood leukocytes by use of a DNA extraction kit (QIAamp® DNA Blood Min Kit, Germany) according to the manufacturer's instructions, and its concentration was determined by using a NanoDrop® ND-1000 Spectrophotometer from Thermo Fisher Scientific Inc. (Waltham, MA, USA). The PCR-restriction fragment length polymorphism (PCR-RFLP) assay was used to analyze the *miR-146a* C > G, *miR-196a2* T > C *miR-499* T > C and *miR-196a2* T > C SNPs. This method was classic, simple and convenient.

PCR primers were designed using PrimerQuestTM (Integrated DNA Technologies, Coralville, IA, USA) according to the reference sequences (*miR-146a*: JX991307.1, *miR-149*: LM608518.1, *miR-196a2*: LM608349.1, *miR-499*: LM609497.1). The PCR of the *miR-146a* C > G SNP was performed using the following primers to generate a 147-bp DNA product: forward: 5′-CAT GGG TTG TGT CAG TGT CAG AGC T-3′ and reverse: 5′-TGC CTT CTG TCT CCA GTC TTC CAA-3′. For the *miR-149* T > C SNP, the following primers were used to generate a 254-bp DNA product: forward: 5′-TGT CTT CAC TCC CGT GCT TGT CC -3′ and reverse: 5′- TGA GGC CCG AAA CAC CCG TA -3′. For the *miR-196a2* T > C SNP, the following primers were used to generate a 149-bp DNA product: forward: 5′-CCC CTT CCC TTC TCC TCC AGA TA -3′ and reverse: 5′-CGA AAA CCG ACT GAT GTA ACT CCG-3′. For the *miR-499* T > C SNP, the following primers were used to amplify a 146-bp DNA fragment: forward: 5′-CAA AGT CTT CAC TTC CCT GCC A-3′ and reverse: 5′-GAT GTT TAA CTC CTC TCC ACG TGA TC-3′.

The PCR reactions were performed in a total volume of 25 μl, including 2.5 μl 10× PCR buffer, 1.5 mmol/l MgCl_2_, 0.15 mmol/l dNTPs, 0.5 μmol/l each primer, 100 ng of genomic DNA and 1 U of Taq DNA polymerase (Takara Bio, Co., Ltd., Dalian, China). The PCR conditions were 94°C for 5 min, followed by 32 cycles of 30 s at 94°C, 30 s at 63°C for the *miR-196a2* T > C SNP, 30 s at 67°C for the *miR-499* T > C SNP, 30 s at 58°C for the *miR-149* T > C SNP, and 30 s at 58°C for the *miR-146a* C > G SNP, and 30 s at 72°C, with a final elongation at 72°C for 10 min.

The *miR-146a* C > G, *miR-149* T > C, *miR-196a2* T > C, and *miR-499* T > C SNPs were detected by digesting the PCR products with restriction endonucleases *SacI*, *PVUII*, *MspI*, and *BclI*, respectively. The endonucleases were purchased from New England Biolabs (Beverly, MA, USA). The PCR products (18 μl) were digested overnight with restriction enzyme. The digested PCR products were separated on a 3.0% agarose gel, stained with ethidium bromide, and visualized under ultraviolet illumination. For *miR-146a* C > G SNP, allele C was cuttable, yielding two fragments of 25 bp and 122 bp, allele G was uncuttable and the fragment was still 147 bp. For *miR-149* T > C SNP allele T was cuttable, yielding two fragments of 60 bp and 194 bp, allele C was uncuttable and the fragment was still 254 bp. For *miR-196a2* T > C SNP, allele C was cuttable, yielding two fragments of 24 bp and 125 bp, allele T was uncuttable and the fragment was still 149 bp. For *miR-499* T > C SNP, allele T was cuttable, yielding two fragments of 26 bp and 120 bp, allele C was uncuttable and the fragment was still 146 bp. For each of the SNPs, 30% of the PCR assays were randomly chosen for a second PCR assay followed by DNA sequencing to validate the RFLP findings. Sequencing was performed using an ABI3730xl DNA Analyzer (Applied Biosystems, Foster City, CA, USA). The concordance rate for the quality control samples was 100%.

### Statistical analysis

Hardy-Weinberg equilibrium (HWE) was assessed by using the chi-square test for each group. The two-tailed Fisher exact method was used to compare allele and genotype distribution in the pulmonary TB patients and healthy controls by the GraphPad Prism version 5.01 software. The odds ratios (OR) and 95% confidence intervals (CI) were calculated by the Miettinen method. The value of OR was used to estimate the relative risk. If the OR is equal to 1, it indicates that the factor has no effect on the incidence of disease. If the OR is greater than 1, it indicates that the factor is a risk to the incidence of disease. If the OR is less than 1, then it indicates that the factor is protective from disease.

The genetic model of SNPs, pairwise linkage disequilibrium (LD) and haplotypes were calculated by using the online SNP analysis software (http://bioinfo.iconcologia.net/SNPstats). Pairwise LD was estimated by calculating pairwise D′ and r^2^ [7,8]. Values of D′ > 0.75 were considered to be strong pairwise LD. Haplotype frequencies and associations were calculated by Haploview version 4.2, which uses the expectation-maximization algorithm. Using logistic regression method, we calculated the allele combinations of OR values and 95% CI referring to the highest frequency of haplotype allele and the additive genetic model. *P* value was calculated to estimate haplotype association with pulmonary TB. A *P* value of < 0.05 was considered to be statistically significant.

## Results

### Analysis of *miR-146a* C > G, *miR-149* T > C, *miR-196a2* T > C and *miR-499* T > C SNPs in the Chinese Uygur population

The *miR-146a* C > G, *miR-149* T > C, *miR-196a2* T > C and *miR-499* T > C SNPs were investigated in 301 pulmonary TB cases and 361 healthy controls in the Chinese Uygur population. All SNPs were in HWE in the control group and the pulmonary TB group (*P* > 0.05) (Table 2).

**Table 2:** **Allele frequencies and genotype distributions of** ***miR-146a*** **C > G,** ***miR-149*** **T > C,** ***miR-196a2*** **T > C and** ***miR-499*** **T > C SNPs in the Chinese Uygur population**

**SNP sites**		**Controls N (Freq)**	**Patients N (Freq)**	***P*** **value**	**OR (95% CI)**
*miR-146a* C > G Allele	G	441(0.611)	358(0.622)	0.693	1 0.96(0.76-1.20)
	C	281(0.389)	218(0.378)		
Genotype	GG	131(0.368)	106(0.368)		1
	GC	179(0.496)	146(0.507)	0.963	1.00(0.72-1.41)
	CC	51(0.141)	36(0.125)	0.591	0.87(0.53-1.43)
	HWE(*P*)	0.415	0.188		
*miR-149* T > C Allele	C	342(0.521)	274(0.511)	0.727	1 1.04(0.83-1.31)
	T	314(0.479)	262(0.489)		
Genotype	CC	89(0.271)	75(0.280)		1
	TC	164(0.500)	124(0.164)	0.581	0.89(0.61-1.32)
	TT	75(0.229)	69(0.257)	0.701	1.09(0.69-1.71)
	HWE(*P*)	0.974	0.225		
*miR-196a2* T > C Allele	C	378(0.531)	286(0.498)	0.244	1 1.14(0.91-1.42)
	T	334(0.469)	288(0.502)		
Genotype	CC	96(0.270)	67(0.233)		1
	TC	186(0.522)	152(0.530)	0.414	1.17(0.80-1.71)
	TT	74(0.208)	68(0.237)	0.234	1.32(0.84-2.07)
	HWE(*P*)	0.356	0.316		
*miR-499* T > C Allele	T	421(0.829)	455(0.756)	0.003	1 1.56(1.16-2.10)
	C	87(0.171)	147(0.244)		
Genotype	TT	171(0.673)	176(0.585)		1
	TC	79(0.311)	103(0.342)	0.199	1.27(0.88-1.82)
	CC	4(0.016)	22(0.073)	0.001	5.34(1.80-15.83)
	HWE(*P*)	0.127	0.205		

SNP: single nucleotide polymorphism, HWE: Hardy-Weinberg equilibrium, OR: odds ratio, CI: confidence interval, N: number of alleles, Freq: frequency. All of the miRNA SNPs were in HWE.

The distribution frequencies of genotypes TT, TC, and CC of the *miR-499* T > C SNP were 0.585, 0.342, and 0.073, respectively, in the control group; while, the frequencies of three genotypes in the pulmonary TB group were 0.673, 0.311, and 0.016, respectively. The frequency of CC genotype in the pulmonary TB group was lower than the control group, and there was a significant difference between the two groups (*P* = 0.001; OR = 5.344; 95% CI, 1.803-15.83). However, there was no significant difference in the frequency of TC genotype between the two groups (*P* > 0.05). No significant differences between pulmonary TB patients and controls were observed in the genotype distribution of *miR-146a* C > G, *miR-149* T > C, and *miR-196a2* T > C SNPs (*P* > 0.05) (Table 2).

The frequency of the allele T of *miR-499* T > C SNP in the pulmonary TB group (0.756) was lower than the control group (0.829), and there was a significant difference between the two groups (*P* = 0.003; OR = 1.563; 95% CI, 1.162-2.103). However, there were no significant differences in allele frequencies of *miR-146a* C > G, *miR-149* T > C, and *miR-196a2* T > C SNPs between the pulmonary TB group and the control group (*P* > 0.05) (Table 2).

We performed logistic regression analysis with all SNPs using a recessive model adjusted for age and sex and observed that the *miR-499* T > C SNP was associated with pulmonary TB in the codominant (*P* = 0.0014; OR = 5.37; 95% CI, 1.81-15.92), dominant (*P* = 0.029; OR = 1.47; 95% CI, 1.04-2.09), recessive (*P* = 7e-04; OR = 4.95; 95% CI, 1.68-14.55), and additive (*P* = 0.0027; OR = 1.57; 95% CI, 1.16-2.11) models. However, the genetic models of *miR-146a* C > G, *miR-149* T > C and *miR-196a2* T > C SNPs did not correlate with pulmonary TB (*P* > 0.05). Moreover, according to the minimum Akaike information criterion (AIC), the best patterns for inheritance of *miR-146a* C > G, *miR-149* T > C, *miR-196a2* T > C and *miR-499* T > C SNPs were recessive model, dominant model, additive model, and codominant model, respectively (see Additional file 1: Table S1).

**Figure 1:**
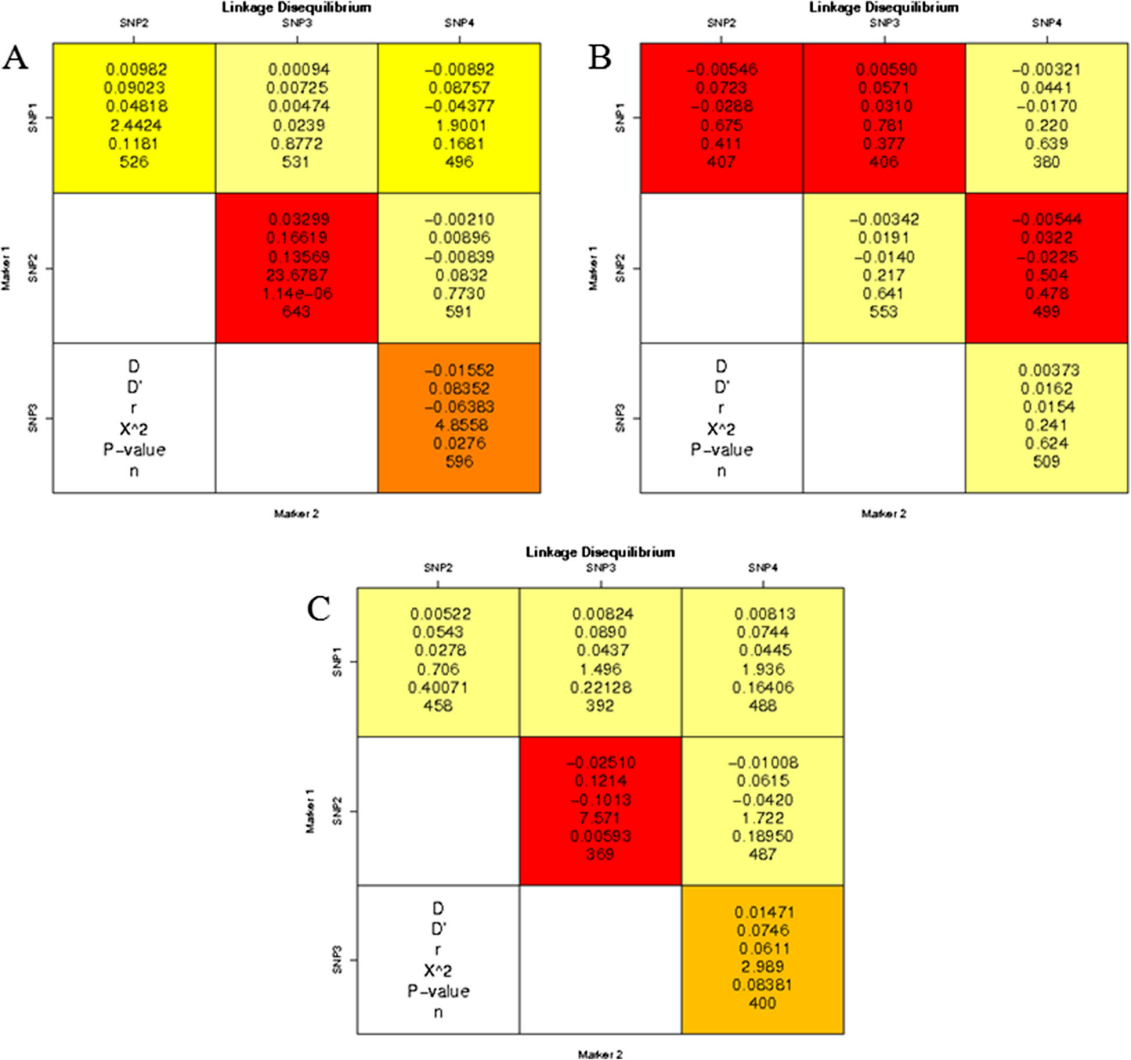
**Linkage disequilibrium analysis of** ***miR-146a*** **C > G,** ***miR-149*** **T > C,** ***miR-196a2*** **T > C, and** ***miR-499*** **T > C SNPs.** The number at the intersection of each pair of SNPs represent the pairwise D, D′, r^2^ and *P* values between two SNPs. Values of D’ > 0.75 and *P* < 0.0001 were considered to be strong pairwise linkage disequilibrium. SNP1: *miR-499*, SNP2: *miR-196a2*, SNP3: *miR-146a*, SNP4: *miR-149*. **A**: Uygur population; **B**: Kazak population; **C**: Southern Han population.

Pairwise LD between *miR-146a* C > G, *miR-149* T > C, *miR-196a2* T > C and *miR-499* T > C SNPs was calculated for the pulmonary TB cases and controls (Figure 1). All pairs of markers in SNPs were not in strong LD (D′< 0.75, *P* < 0.0001). Therefore, based on logistic regression, the haplotypes for each pair of SNPs were constructed. The frequency of the haplotype CTCC in the control group was lower than the pulmonary TB group, and was significantly associated with pulmonary TB (*P* = 0.044; OR = 7.19; 95% CI, 1.01-51.14). However, other haplotypes showed no significant association with pulmonary TB (*P* > 0.05) (see Additional file 1: Table S2).

### Analysis of *miR-146a* C > G, *miR-149* T > C, *miR-196a2* T > C and *miR-499* T > C SNPs in the Chinese Kazak population

The *miR-146a* C > G, *miR-149* T > C, *miR-196a2* T > C, and *miR-499* T > C SNPs were investigated in 251 pulmonary TB cases and 362 healthy controls in the Chinese Kazak population. All SNPs were in HWE in the control group and the pulmonary TB group (*P* > 0.05) (Table 3).

**Table 3:** **Allele frequencies and genotype distributions of** ***miR-146a*** **C > G,** ***miR-149*** **T > C,** ***miR-196a2*** **T > C and** ***miR-499*** **T > C SNPs in the Chinese Kazak population**

**SNP sites**		**Controls N (Freq)**	**Patients N (Freq)**	***P*** **value**	**OR (95% CI)**
*miR-146a* C > G Allele	G	422(0.598)	284(0.402)	0.023	1 1.33(1.04-1.69)
	C	222(0.529)	198(0.171)		
Genotype	GG	124(0.351)	65(0.310)		1
	GC	174(0.493)	92(0.438)	0.965	1.01(0.68-1.49)
	CC	55(0.156)	53(0.252)	0.013	1.84(1.14-2.98)
	HWE(*P*)	0.637	0.080		
*miR-149* T > C Allele	T	437(0.604)	189(0.580)	0.466	1 1.10(0.84-1.44)
	C	287(0.396)	137(0.420)		
Genotype	TT	131(0.362)	52(0.319)		1
	TC	175(0.483)	85(0.521)	0.338	1.22 (0.81-1.85)
	CC	56(0.155)	26(0.160)	0.587	1.17 (0.66-2.06)
	HWE(*P*)	0.845904	0.370294		
*miR-196a2* T > C Allele	C	378(0.549)	314(0.625)	0.008	1 0.73(0.58-0.92)
	T	310(0.451)	188(0.451)		
Genotype	CC	103(0.299)	99(0.394)		1
	TC	172(0.500)	116(0.462)	0.055	0.72(0.49-1.01)
	TT	69(0.201)	36(0.143)	0.013	0.54(0.33-0.88)
	HWE(*P*)	0.855	0.830		
*miR-499* T > C Allele	T	382(0.813)	285(0.828)	0.565	10.90(0.62-1.29)
	C	88(0.187)	59(0.172)		
Genotype	TT	157(0.668)	116(0.674)		1
	TC	68(0.298)	53(0.308)	0.808	1.06(0.68-1.62)
	CC	10(0.043)	3(0.017)	0.165	0.41(0.11-1.51)
	HWE(*P*)	0.450	0.269		

SNP: single nucleotide polymorphism, HWE: Hardy-Weinberg equilibrium, OR: odds ratio, CI: confidence interval, N: number of alleles, Freq: frequency. All of the miRNA SNPs were in HWE.

The distribution frequencies of genotypes GG, GC, and CC of the *miR-146a* C > G SNP were 0.351, 0.493, and 0.156, respectively, in the control group; while, the frequencies of the three genotypes in the pulmonary TB group were 0.310, 0.438, and 0.252, respectively. The frequency of CC genotype in the pulmonary TB group was lower than the control group, and there was a significant difference between the two groups (*P* = 0.013; OR = 1.84; 95% CI, 1.14-2.98). However, there was no significant difference in the frequency of GC genotype between the two groups (*P* > 0.05). The distribution frequencies of genotypes CC, TC, and TT of the *miR-196a2* T > C SNP in were 0.299, 0.500, and 0.201, respectively, the control group; while, the frequencies of the three genotypes in the pulmonary TB group were 0.394, 0.462, and 0.143, respectively. The frequency of TT genotype in the pulmonary TB group was higher than the control group, and there was a significant difference between the two groups (*P* = 0.013; OR = 0.54; 95% CI, 0.33-0.88). However, there was no significant difference in the frequency of TC genotype between the two groups (*P* > 0.05). No significant differences between pulmonary TB patients and healthy controls were observed in the genotype distribution of *miR-149* T > C and *miR-499* T > C SNPs (*P* > 0.05) (Table 3).

The frequency of the allele G of *miR-146a* C > G SNP in the pulmonary TB group (0.402) was lower than the control group (0.598), and there was a significant difference between the two groups (*P* = 0.023; OR = 1.33; 95% CI, 1.04-1.69). The frequency of the allele C of *miR-196a2* T > C SNP in the pulmonary TB group (0.625) was higher than the control group (0.549), and there was a significant difference between the two groups (*P* = 0.008; OR = 0.73; 95% CI, 0.58-0.92). However, no significant differences were observed in the allele frequencies of *miR-149* T > C and *miR-499* T > C SNPs between the pulmonary TB group and the control group (*P* > 0.05) (Table 3).

We performed logistic regression analysis with all SNPs using a recessive model adjusted for age and sex and observed that the *miR-146a* C > G SNP was associated with pulmonary TB in the codominant (*P* = 0.019; OR = 1.87; 95% CI, 1.15-3.03), recessive (*P* = 0.005; OR = 1.84; 95% CI, 1.20-2.81), and additive (*P* = 0.022; OR = 1.33; 95% CI, 1.04-1.69) models. The *miR-196a2* T > C SNP was found to be the codominant model (*P* = 0.029; OR = 0.54; 95% CI, 0.33-0.88), dominant model (*P* = 0.014; OR = 0.65; 95% CI, 0.46-0.92), and additive model (*P* = 0.0083; OR = 0.73; 95% CI, 0.58-0.92) in association with pulmonary TB. However, the genetic models of *miR-149* T > C and *miR-499* T > C SNPs did not correlate with pulmonary TB (*P* > 0.05). Moreover, according to the minimum AIC, the best patterns for inheritance of *miR-146a* C > G, *miR-149* T > C, *miR-196a2* T > C and *miR-499* T > C SNPs were recessive model, dominant model, additive model, and recessive model, respectively (see Additional file 1: Table S3).

Pairwise LD between *miR-146a* C > G, *miR-149* T > C, *miR-196a2* T > C and *miR-499* T > C SNPs was calculated for the pulmonary TB cases and controls (Figure 1). All pairs of markers in SNPs were not in strong LD (D′< 0.75, *P* < 0.0001). Therefore, based on logistic regression, the haplotypes for each pair of SNPs were constructed. However, all haplotypes showed no significant association with pulmonary TB (*P* > 0.05) (see Additional file 1: Table S4).

### Analysis of *miR-146a* C > G, *miR-149* T > C, *miR-196a2* T > C, and *miR-499* T > C SNPs in the Chinese Southern Han population

The *miR-146a* C > G, *miR-149* T > C, *miR-196a2* T > C, and *miR-499* T > C SNPs were investigated in 354 pulmonary TB cases and 300 healthy controls in the Chinese Southern Han population. All SNPs were in HWE in the control group and the pulmonary TB group (*P* > 0.05) (see Additional file 1: Table S5). No significant differences were observed in the genotype distribution of *miR-146a* C > G, *miR-149* T > C, *miR-196a2* T > C and *miR-499* T > C SNPs between the pulmonary TB group and the control group (*P* > 0.05) (see Additional file 1: Table S5).

There was no significant difference in the allele frequencies of *miR-146a* C > G, *miR-149* T > C *miR-196a2* T > C, and *miR-499* T > C SNPs between pulmonary TB patients and controls (*P* > 0.05) (see Additional file 1: Table S5).

We performed logistic regression analysis with the *miR-146a* C > G, *miR-149* T > C, *miR-196a2* T > C and *miR-499* T > C SNPs using a recessive model adjusted for age and sex. The genetic models of *miR-146a* C > G, *miR-196a2* T > C, *miR-149* T > C, and *miR-499* T > C SNPs did not correlate with pulmonary TB (*P* > 0.05). Moreover, according to the minimum AIC, the best patterns for inheritance of *miR-146a* C > G, *miR-149* T > C, *miR-196a2* T > C and *miR-499* T > C SNPs were all additive model (see Additional file 1: Table S6).

Pairwise LD between *miR-146a* C > G, *miR-149* T > C, *miR-196a2* T > C, and *miR-499* T > C SNPs was calculated for the pulmonary TB cases and controls (Figure 1). All pairs of markers in SNPs were not in strong LD (D′ < 0.75, *P* < 0.0001). Therefore, based on logistic regression, the haplotypes for each pair of SNPs were constructed. The frequency of the haplotype TCCC in the control group was higher than the pulmonary TB group, and was significantly associated with pulmonary TB (*P* = 0.044; OR = 0.23; 95% CI, 0.05 - 0.96). The frequency of the haplotype CCCT in the control group was higher than the pulmonary TB group, and was significantly associated with pulmonary TB (*P* = 0.024; OR = 0.03; 95% CI, 0.01 - 0. 63) (Table 4).

**Table 4:** **Haplotype frequencies of** ***miR-146a*** **C > G,** ***miR-149*** **T > C,** ***miR-196a2*** **T > C and** ***miR-499*** **T > C SNPs in the Southern Han population**

**Haplotype**	**Allele at marker**				**Controls N (Freq)**	**Patients N (Freq)**	**Total N (Freq)**	**OR (95% CI)**	***P*** **value**
	***miR-499***	***miR-196a2***	***miR-146a***	***miR-149***					
1	T	C	C	T	0.1639	0.1699	0.1643	1.00	---
2	T	T	G	T	0.1491	0.1689	0.1487	0.96 (0.51 - 1.79)	0.9
3	T	T	C	T	0.1266	0.136	0.1378	0.67 (0.27 - 1.65)	0.38
4	T	T	C	C	0.0662	0.1378	0.0953	2.33 (0.98 - 5.55)	0.057
5	T	T	G	C	0.0761	0.0697	0.0828	0.60 (0.24 - 1.50)	0.27
6	T	C	G	T	0.0926	0.0533	0.0796	0.35 (0.10 - 1.19)	0.092
7	T	C	G	C	0.0711	0.0736	0.0652	0.95 (0.38 - 2.36)	0.91
8	T	C	C	C	0.0607	0.0358	0.053	0.23 (0.05 - 0.96)	0.044
9	C	T	G	T	0.0384	0.0174	0.0366	0.24 (0.04 - 1.37)	0.11
10	C	C	C	T	0.0325	0.0065	0.0303	0.03 (0.01 - 0.63)	0.024
11	C	T	C	T	0.0182	0.0365	0.0211	3.38 (0.54 - 21.22)	0.19
12	C	C	C	C	0.0333	0.0188	0.0194	0.42 (0.04 - 4.04)	0.45
13	C	C	G	C	0	0.0244	0.0189	0.00 (-Inf - Inf)	1
14	C	T	G	C	0.0457	0	0.0181	0.00 (-Inf - Inf)	1
15	C	T	C	C	0.0052	0.017	0.0158	1.41 (0.02 - 90.56)	0.87
16	C	C	G	T	0.0203	0.0344	0.0131	1.35 (0.19 - 9.60)	0.76
Global haplotype association *P*-value: 0.0084									

Freq: frequency of haplotype, OR: odds ratio, N: number of subjects, CI: confidence interval.

## Discussion

In our study, miRNAs were screened and identified as novel potential combination biomarkers for pulmonary TB [24]. Specific miRNAs have been shown to be associated with pulmonary TB previously [20-22]. It was suggested that miRNAs may be involved in the pathogenesis of pulmonary TB and play an important role in *Mtb* infection. The first study that identified SNPs in precursor miRNAs was performed in 2005 [25]. The researches on small molecule RNA will provide a new method to understand the pathogenesis and treatment of pulmonary TB.

No associations so far has been reported between *miR-146a* C > G, *miR-149* T > C, *miR-196a2* T > C and *miR-499* T > C SNPs and susceptibility to pulmonary TB in the Chinese Uygur, Kazak, and Southern Han populations. We speculated that inconsistencies may exist between the four SNPs and susceptibility to pulmonary TB in these populations. In the Uygur population, there was a significant difference in the frequency of allele T and CC genotype of the *miR-499* T > C SNP between the pulmonary TB patients and healthy controls. The OR values were more than 1, indicating that the *miR-499* T > C SNP can be associated with susceptibility to pulmonary TB in the Uygur population and may increase the risk of being infected with pulmonary TB. There were no significant differences in the frequencies of alleles and genotypes of *miR-146a* C > G, *miR-149* T > C and *miR-196a2* T > C SNPs between pulmonary TB patients and healthy controls, indicating that these SNPs may not be associated with susceptibility to pulmonary TB in the Uygur population. However, in the Kazak population, there was a significant difference in the frequency of allele G and CC genotype of the *miR-146a* C > G SNP between the pulmonary TB group and the control group. The OR values were more than 1, indicating that the *miR-146a* C > G SNP can be associated with susceptibility to pulmonary TB in the Kazak population and may increase the risk of being infected with pulmonary TB. There was a significant difference in the frequency of allele C and TT genotype of the *miR-196a2* T > C SNP between the pulmonary TB group and the control group, indicating that the *miR-196a2* T > C SNP can be associated with pulmonary TB in the Kazak population. However, no significant differences were observed in the allele and genotype distributions of *miR-149* T > C and *miR-499* T > C SNPs between pulmonary TB patients and controls, indicating that the *miR-196a2* T > C SNP may not be associated with susceptibility to pulmonary TB in the Kazak population. There were no significant differences in the allele and genotype distributions of *miR-146a* C > G, *miR-149* T > C, *miR-196a2* T > C, and *miR-499* T > C SNPs between the pulmonary TB group and the control group in the Southern Han population, indicating that the four SNPs did not correlate with susceptibility to pulmonary TB in this population. Our study is the first to report the association between *miR-146a*, *miR-149*, *miR-196a2* and *miR-499* SNPs and susceptibility to pulmonary TB in the Chinese Uygur, Kazak, and Southern Han populations. The results confirmed that the frequencies of susceptibility to pulmonary TB in the Uygur and Kazak populations were higher than the Southern Han population in China [2].

LD was also calculated between *miR-146a* C > G, *miR-149* T > C, *miR-196a2* T > C, and *miR-499* T > C SNPs. In the Uygur population, the frequency of haplotype CTCC was significantly associated with pulmonary TB and may increase the risk of pulmonary TB infection. However, in the Kazak population, all haplotypes showed no significant association with pulmonary TB. In the Southern Han population, the haplotype frequencies of TCCC and CCCT were found to be significantly associated with pulmonary TB. The OR values were less than 1, indicating reduced risk of being infected with pulmonary TB. Therefore, we proposed that SNPs caused by various ethnic groups may affect susceptibility to pulmonary TB. Meanwhile, gene-gene interaction often combine with genetic and environmental factors, and make the observed difference even more complicated between different ethnic groups.

Li et al. [26] reported the association of *miR-146a* C > G and *miR-499* T > C SNPs with susceptibility to pulmonary TB in the Chinese Tibetan and Han populations. The study found significant differences in the allele and genotype frequencies of the *miR-146a* C > G SNP between the pulmonary TB patients and healthy controls. The OR values were more than 1, indicating that the *miR-146a* C > G SNP can be associated with susceptibility to pulmonary TB in the Tibetan population. There were significant differences in the allele and genotype frequencies of the *miR-146a* C > G SNP between the pulmonary TB patients and healthy controls. The OR values were less than 1, indicating that the *miR-146a* C > G SNP can be associated with pulmonary TB and may reduce the risk of being infected with pulmonary TB in a Chinese Han population. The results of the present study showed that the *miR-146a* C > G SNP was not associated with susceptibility to pulmonary TB in the Chinese Uygur and Southern Han populations. However, the *miR-146a* C > G SNP was associated with susceptibility to pulmonary TB in the Chinese Kazak population. According to Hap Map Organization statistics, different ethnic groups showed significant differences in the distribution frequency of SNPs. We speculated that this is the main reason for differences in different ethnic groups. Our study provides a theoretical basis for genetic epidemiological research in different ethnic groups [27]. The *miR-146a* C > G SNP is located in the stem region opposite to the mature sequence and lead to a change from G:U pair to C:U mismatch in miR-146a precursor [26]. MiR-146a plays an important role in the regulation of toll-like receptors (TLRs), cytokine, and NF-κB signaling pathway [28]. NF-κB is a key molecule in the innate immune system, because it not only promotes recognition and clearance of pathogens, such as *Mtb*, but also involves the transcription of many genes related to immune responses that could limit the spread of invading pathogens [29]. Several experimental studies have provided evidence that the allele C in the *miR-146a* C > G SNP could reduce mature miR-146a production, which might modify the inflammatory process [30]. Although the role of *miR-146a* C > G SNP in the pathogenesis of pulmonary TB is unclear, this SNP may interfere with *Mtb* infection in immune response, thereby increasing the occurrence of pulmonary TB.

Li et al. [26] found significant differences in the allele and genotype frequencies of the *miR-499* T > C SNP between the pulmonary TB patients and healthy controls. The OR values were more than 1, indicating that the *miR-499* T > C SNP can be associated with susceptibility to pulmonary TB in the Chinese Tibetan population. There was no significant difference in the allele frequency of the *miR-499* T > C SNP between the pulmonary TB patients and healthy controls in the Chinese Han population. However, significant differences were observed in the genotype frequencies of the *miR-499* T > C SNP between the pulmonary TB patients and healthy controls. The OR values were less than 1, indicating that the *miR-499* T > C SNP can be associated with pulmonary TB and may reduce the risk of being infected with TB in the Chinese Han population. The present study showed that the *miR-499* T > C SNP was associated with susceptibility to pulmonary TB in the Chinese Uygur population, but not Chinese Kazak and Southern Han populations. We speculated that individual differences in the genetic background may lead to differences between SNPs and susceptibility to disease. The *miR-499* T > C SNP is located in the 3p strand in mature miRNA regions. Also, the *miR-499* T > C SNP might affect the binding of target mRNAs to 3p mature miRNAs, and may alter the function of miR-499 [31].

This study is the first to report that *miR-149* T > C and *miR-196a2* T > C SNPs are correlated with pulmonary TB. The results showed that the *miR-196a2* T > C SNP was associated with susceptibility to pulmonary TB in the Chinese Uygur and Southern Han populations, but not the Chinese Kazak population. Hu et al. [32] found that the genotype CC of the *miR-196a2* T > C SNP was related to the high expression levels of mature miR-196a. Therefore, in the Chinese Kazak population, the genotype CT or TT of the *miR-196a2* T > C SNP decreased the expression of mature miR-196a, and may be involved in the pathogenesis of pulmonary TB. The *miR-149* T > C SNP was not associated with pulmonary TB in the Chinese Uygur, Kazak, and Southern Han populations, indicating that the *miR-149* T > C SNP may not be correlated with susceptibility to pulmonary TB. This also confirms our hypothesis.

Analysis of alleles, genotypes and haplotypes frequencies and genetic patterns revealed that the *miR-499* T > C SNP can be associated with susceptibility to pulmonary TB in the Chinese Uygur population and may increase the risk of being infected with TB. However, *miR-146a* C > G, *miR-149* T > C, and *miR-196a2* T > C SNPs were not associated with pulmonary TB in the Chinese Uygur population. *MiR-146a* C > G and *miR-196a2* T > C SNPs were associated with pulmonary TB in the Chinese Kazak population, but no such associations were found in *miR-149* T > C and *miR-499* T > C SNPs. *MiR-146a* C > G, *miR-149* T > C, *miR-196a2* T > C, and *miR-499* T > C SNPs may not be associated with susceptibility to pulmonary TB in the Chinese Southern Han population. The present study explored the pathogenesis of pulmonary TB from the perspective of miRNA SNPs, and provided a new idea to distinguish high-risk populations with pulmonary TB in order to prevent and control pulmonary TB transmission.

## Conclusions

*MiR-146a*, *miR-149*, *miR-196a2,* and *miR-499* SNPs can be associated with susceptibility to pulmonary TB in the Chinese Uygur, Kazak, and Southern Han populations. It is therefore suggested that susceptibility to pulmonary TB may be closely related to individual differences caused by genetic factors in a Chinese population.

## Additional file

Additional file 1: Table S1. Association of *miR-146a* C > G, *miR-149* T > C, *miR-196a2* T > C and *miR-499* T > C SNPs with pulmonary TB in the Chinese Uygur population using the logistic regression model. **Table S2.** Haplotype frequencies of *miR-146a* C > G, *miR-149* T > C, *miR-196a2* T > C and *miR-499* T > C SNPs in the Chinese Uygur population (patients with pulmonary TB and healthy controls). **Table S3.** Association of *miR-146a* C > G, *miR-149* T > C, *miR-196a2* T > C and *miR-499* T > C SNPs with pulmonary TB in the Chinese Kazak population using the logistic regression model. **Table S4.** Haplotype frequencies of *miR-146a* C > G, *miR-149* T > C, *miR-196a2* T > C and *miR-499* T > C SNPs in the Chinese Kazak population (patients with pulmonary TB and healthy controls). **Table S5.** Allele frequencies and genotype distributions of *miR-146a* C > G, *miR-149* T > C, *miR-196a2* T > C and *miR-499* T > C SNPs in the Southern Han population (pulmonary TB and control groups). **Table S6.** Association of *miR-146a* C > G, *miR-149* T > C, *miR-196a2* T > C and *miR-499* T > C SNPs with pulmonary TB in the Southern Han population using the logistic regression model.

## Abbreviations

SNPs: Single nucleotide polymorphisms
miRNAs: microRNAs
TB: Pulmonary tuberculosis
Mtb: Mycobacterium tuberculosis
WHO: World health organization
TLR2: Toll-like receptor 2
NOD2: NOD-like receptor 2
MRC1: Mannose receptor 1
CTLA4: Cytotoxic T lymphocyte antigen-4
pre-miRNAs: precursor miRNAs
HWE: Hardy-Weinberg equilibrium
OR: Odd ratio
CI: Confidence interval
LD: Linkage disequilibrium
TLRs: Toll-like receptors
