# Supplementary material for: Association of the miR-146a, miR-149, miR-196a2 and miR-499 polymorphisms with susceptibility to pulmonary tuberculosis in the Chinese Uygur, Kazak and Southern Han populations
Source: BMC Infect Dis. 2015 Feb 5;15:41. doi: 10.1186/s12879-015-0771-9 (PMC4326450; doi:10.1186/s12879-015-0771-9)
Supplement: Additional file 1: Table S1. — Association of miR-146a C > G, miR-149 T > C, miR-196a2 T > C and miR-499 T > C SNPs with pulmonary TB in the Chinese Uygur population using the logistic regression model. Table S2. Haplotype frequencies of miR-146a C > G, miR-149 T > C, miR-196a2 T > C and miR-499 T > C SNPs in the Chinese Uygur population (patients with pulmonary TB and healthy controls). Table S3. Association of miR-146a C > G, miR-149 T > C, miR-196a2 T > C and miR-499 T > C SNPs with pulmonary TB in the Chinese Kazak population using the logistic regression model. Table S4. Haplotype frequencies of miR-146a C > G, miR-149 T > C, miR-196a2 T > C and miR-499 T > C SNPs in the Chinese Kazak population (patients with pulmonary TB and healthy controls). Table S5. Allele frequencies and genotype distributions of miR-146a C > G, miR-149 T > C, miR-196a2 T > C and miR-499 T > C SNPs in the Southern Han population (pulmonary TB and control groups). Table S6. Association of miR-146a C > G, miR-149 T > C, miR-196a2 T > C and miR-499 T > C SNPs with pulmonary TB in the Southern Han population using the logistic regression model. [file 12879_2015_771_MOESM1_ESM.docx]

**Additional file 1: Table S1.** Association of *miR-146a* C > G, *miR-149* T > C, *miR-196a2* T > C and *miR-499* T > C SNPs with pulmonary TB in a Chinese Uygur population using logistic regression

| **Model^a^** | **Genotype** | **Controls** | **Patients** | **OR (95% CI)** | ***P* value** | **AIC** | **BIC** |
| --- | --- | --- | --- | --- | --- | --- | --- |
| *miR-146a* C > G Codominant | G/G  G/C  C/C | 131 (36.3%)  179 (49.6%)  51 (14.1%) | 106 (36.9%)  145 (50.5%)  36 (12.5%) | 1.00  1.00 (0.71-1.40)  0.87 (0.53-1.43) | 0.84 | 895.5 | 908.9 |
| Dominant | G/G  G/C-C/C | 131 (36.3%)  230 (63.7%) | 106 (36.9%)  181 (63.1%) | 1.00  0.97 (0.71-1.34) | 0.87 | 893.8 | 902.8 |
| Recessive | G/G-G/C  C/C | 310 (85.9%)  51 (14.1%) | 251 (87.5%)  36 (12.5%) | 1.00  0.87 (0.55-1.38) | 0.56 | 893.5 | 902.5 |
| Overdominant | G/G- C/C  G/C | 182 (50.4%)  179 (49.6%) | 142 (49.5%)  145 (50.5%) | 1.00  1.04 (0.76-1.42) | 0.81 | 893.8 | 902.7 |
| Log-additive | --- | --- | --- | 0.95 (0.75-1.20) | 0.67 | 893.7 | 902.6 |
| *miR-149* T > C Codominant | C/C  T/C  T/T | 75 (28.1%)  123 (46.1%)  69 (25.8%) | 89 (27.1%)  164 (50%)  75 (22.9%) | 1.00  1.12 (0.76-1.65)  0.92 (0.58-1.43) | 0.59 | 823.5 | 836.7 |
| Dominant | C/C  T/C-T/T | 75 (28.1%)  192 (71.9%) | 89 (27.1%)  239 (72.9%) | 1.00  1.05 (0.73-1.51) | 0.8 | 822.5 | 831.3 |
| Recessive | C/C-T/C  T/T | 198 (74.2%)  69 (25.8%) | 253 (77.1%)  75 (22.9%) | 1.00  0.85 (0.58-1.24) | 0.4 | 821.9 | 830.6 |
| Overdominant | C/C-T/T  T/C | 144 (53.9%)  123 (46.1%) | 164 (50%)  164 (50%) | 1.00  1.17 (0.85-1.62) | 0.34 | 821.7 | 830.4 |
| Log-additive | --- | --- | --- | 0.96 (0.77-1.20) | 0.73 | 822.9 | 831.6 |
| *miR-196a2* T > C Codominant | C/C  T/C  T/T | 96 (27%)  186 (52.2%)  74 (20.8%) | 66 (23.1%)  152 (53.1%)  68 (23.8%) | 1.00  1.19 (0.81-1.74)  1.34 (0.85-2.11) | 0.44 | 886.7 | 900.1 |
| Dominant | C/C  T/C-T/T | 96 (27%)  260 (73%) | 66 (23.1%)  220 (76.9%) | 1.00  1.23 (0.86-1.77) | 0.26 | 885.1 | 894 |
| Recessive | C/C-T/C  T/T | 282 (79.2%)  74 (20.8%) | 218 (76.2%)  68 (23.8%) | 1.00  1.19 (0.82-1.73) | 0.36 | 885.5 | 894.5 |
| Overdominant | C/C-T/T  T/C | 170 (47.8%)  186 (52.2%) | 134 (46.9%)  152 (53.1%) | 1.00  1.04 (0.76-1.42) | 0.82 | 886.3 | 895.2 |
| Log-additive | --- | --- | --- | 1.16 (0.92-1.45) | 0.21 | 884.8 | 893.7 |
| *miR-499* T > C Codominant | T/T  T/C  C/C | 171 (67.3%)  79 (31.1%)  4 (1.6%) | 175 (58.3%)  103 (34.3%)  22 (7.3%) | 1.00  1.27 (0.89-1.83)  5.37 (1.81-15.92) | 0.0014 | 757.1 | 770 |
| Dominant | T/T  T/C-C/C | 171 (67.3%)  83 (32.7%) | 175 (58.3%)  125 (41.7%) | 1.00  1.47 (1.04-2.09) | 0.029 | 763.4 | 772.1 |
| Recessive | T/T-T/C  C/C | 250 (98.4%)  4 (1.6%) | 278 (92.7%)  22 (7.3%) | 1.00  4.95 (1.68-14.55) | 7e-04 | 756.8 | 765.4 |
| Overdominant | T/T-C/C  T/C | 175 (68.9%)  79 (31.1%) | 197 (65.7%)  103 (34.3%) | 1.00  1.16 (0.81-1.65) | 0.42 | 767.5 | 776.2 |
| Log-additive | --- | --- | --- | 1.57 (1.16-2.11) | 0.0027 | 759.2 | 767.8 |

^a^Adjusted for age and sex. OR: odds ratios, CI: confidence intervals, AIC: Akaike’s Information Criterion, BIC: Bayesian Information Criterion.

**Table S2.** Haplotype frequencies of the *miR-146a* C > G, *miR-149* T > C, *miR-196a2* T > C and *miR-499* T > C SNPs in patients with pulmonary TB and healthy controls in a Chinese Uygur population

| **Haplotype** | **Allele at marker** | | | | **Controls**  **N(Freq)** | **Patients N(Freq)** | **Total**  **N(Freq)** | **OR (95% CI)** | ***P* value** |
| --- | --- | --- | --- | --- | --- | --- | --- | --- | --- |
|  | ***miR-499*** | ***miR-196a2*** | ***miR-146a*** | ***miR-149*** |  |  |  |  |  |
| 1  2  3  4  5  6  7  8  9  10  11  12  13  14  15  16 | T  T  T  T  T  T  T  T  C  C  C  C  C  C  C  C | C  C  T  T  T  C  T  C  C  T  C  T  T  T  C  C | G  G  G  G  C  C  C  C  G  C  G  G  G  C  C  C | T  C  T  C  C  C  T  T  T  C  C  C  T  T  C  T | 0.1472  0.145  0.1057  0.108  0.0935  0.073  0.0836  0.0716  0.0339  0.0106  0.0331  0.0315  0.0063  0.0296  0.0253  0.002 | 0.1373  0.1101  0.1192  0.0988  0.0724  0.0989  0.0684  0.0501  0.0428  0.0581  0.0447  0.029  0.0394  0.0157  0  0.0149 | 0.1403  0.1359  0.1091  0.0999  0.0837  0.0793  0.0785  0.0621  0.0395  0.0363  0.035  0.0318  0.0241  0.0203  0.0145  0.0099 | 1.00  0.84 (0.43 - 1.64)  1.16 (0.58 - 2.35)  1.05 (0.54 - 2.06)  0.64 (0.27 - 1.50)  1.42 (0.70 - 2.88)  0.96 (0.41 - 2.22)  0.81 (0.27 - 2.42)  1.25 (0.40 - 3.87)  7.19 (1.01 - 51.14)  1.21 (0.41 - 3.56)  1.10 (0.31 - 3.85)  4.63 (0.63 - 33.78)  0.55 (0.04 - 7.00)  0.00 (-Inf - Inf)  4.78 (0.00 - 659208.54) | ---  0.6  0.67  0.88  0.3  0.32  0.92  0.7  0.7  0.044  0.74  0.89  0.13  0.64  1  0.8 |
| Global haplotype association *P*-value: 0.18 | | | | | | | | | |

Freq: frequency of haplotype, OR: odds ratios, CI: confidence intervals.

**Table S3.** Association of *miR-146a* C > G, *miR-149* T > C, *miR-196a2* T > C and *miR-499* T > C SNPs with pulmonary TB in a Chinese Kazak population using logistic regression

| **Model^a^** | **Genotype** | **Controls** | **Patients** | **OR (95% CI)** | ***P* value** | **AIC** | **BIC** |
| --- | --- | --- | --- | --- | --- | --- | --- |
| *miR-146a* C > G Codominant | G/G  G/C  C/C | 124 (35.1%)  174 (49.3%)  55 (15.6%) | 64 (30.6%)  92 (44%)  53 (25.4%) | 1.00  1.02 (0.69-1.52)  1.87 (1.15-3.03) | 0.019 | 739.9 | 752.9 |
| Dominant | G/G  G/C-C/C | 124 (35.1%)  229 (64.9%) | 64 (30.6%)  145 (69.4%) | 1.00  1.23 (0.85-1.77) | 0.27 | 744.6 | 753.2 |
| Recessive | G/G-G/C  C/C | 298 (84.4%)  55 (15.6%) | 156 (74.6%)  53 (25.4%) | 1.00  1.84 (1.20-2.81) | 0.005 | 737.9 | 746.6 |
| Overdominant | G/G-C/C  G/C | 179 (50.7%)  174 (49.3%) | 117 (56%)  92 (44%) | 1.00  0.81 (0.57-1.14) | 0.23 | 744.3 | 753 |
| Log-additive | --- | --- | --- | 1.33 (1.04-1.69) | 0.022 | 740.5 | 749.2 |
| *miR-149* T > C Codominant | T/T  T/C  C/C | 131 (36.2%)  175 (48.3%)  56 (15.5%) | 51 (31.5%)  85 (52.5%)  26 (16.1%) | 1.00  0.80 (0.53-1.21)  0.84 (0.48-1.48) | 0.57 | 653 | 665.8 |
| Dominant | T/T  T/C-C/C | 131 (36.2%)  231 (63.8%) | 51 (31.5%)  111 (68.5%) | 1.00  0.81 (0.55-1.20) | 0.29 | 651 | 659.5 |
| Recessive | T/T-T/C  C/C | 306 (84.5%)  56 (15.5%) | 136 (84%)  26 (16.1%) | 1.00  0.96 (0.58-1.59) | 0.87 | 652.1 | 660.6 |
| Overdominant | T/T-C/C  T/C | 187 (51.7%)  175 (48.3%) | 77 (47.5%)  85 (52.5%) | 1.00  0.85 (0.59-1.23) | 0.38 | 651.3 | 659.9 |
| Log-additive | --- | --- | --- | 0.89 (0.68-1.17) | 0.41 | 651.4 | 660 |
| *miR-196a2* T > C Codominant | C/C  T/C  T/T | 103 (29.9%)  172 (50%)  69 (20.1%) | 99 (39.6%)  115 (46%)  36 (14.4%) | 1.00  0.70 (0.48-1.00)  0.54 (0.33-0.88) | 0.029 | 807.4 | 820.6 |
| Dominant | C/C  T/C-T/T | 103 (29.9%)  241 (70.1%) | 99 (39.6%)  151 (60.4%) | 1.00  0.65 (0.46-0.92) | 0.014 | 806.5 | 815.3 |
| Recessive | C/C-T/C  T/T | 275 (79.9%)  69 (20.1%) | 214 (85.6%)  36 (14.4%) | 1.00  0.67 (0.43-1.04) | 0.072 | 809.3 | 818.1 |
| Overdominant | C/C-T/T  T/C | 172 (50%)  172 (50%) | 135 (54%)  115 (46%) | 1.00  0.85 (0.61-1.18) | 0.34 | 811.6 | 820.4 |
| Log-additive | --- | --- | --- | 0.73 (0.58-0.92) | 0.0083 | 805.5 | 814.3 |
| *miR-499* T > C Codominant | T/T  T/C  C/C | 157 (66.8%)  68 (28.9%)  10 (4.3%) | 115 (67.2%)  53 (31%)  3 (1.8%) | 1.00  1.06 (0.69-1.64)  0.41 (0.11-1.52) | 0.33 | 556.5 | 568.5 |
| Dominant | T/T  T/C-C/C | 157 (66.8%)  78 (33.2%) | 115 (67.2%)  56 (32.8%) | 1.00  0.98 (0.64-1.49) | 0.93 | 556.7 | 564.7 |
| Recessive | T/T-T/C  C/C | 225 (95.7%)  10 (4.3%) | 168 (98.2%)  3 (1.8%) | 1.00  0.40 (0.11-1.48) | 0.14 | 554.6 | 562.6 |
| Overdominant | T/T-C/C  T/C | 167 (71.1%)  68 (28.9%) | 118 (69%)  53 (31%) | 1.00  1.10 (0.72-1.69) | 0.65 | 556.5 | 564.5 |
| Log-additive | --- | --- | --- | 0.90 (0.63-1.30) | 0.59 | 556.4 | 564.4 |

^a^Adjusted for age and sex. OR: odds ratios, CI: confidence intervals, AIC: Akaike’s Information Criterion, BIC: Bayesian Information Criterion.

**Table S4.** Haplotype frequencies of the *miR-146a* C > G, *miR-149* T > C, *miR-196a2* T > C and *miR-499* T > C SNPs in patients with pulmonary TB and healthy controls in a Chinese Kazak population

| **Haplotype** | **Allele at marker** | | | | **Controls**  **N(Freq)** | **Patients N(Freq)** | **Total**  **N(Freq)** | **OR (95% CI)** | ***P* value** |
| --- | --- | --- | --- | --- | --- | --- | --- | --- | --- |
|  | ***miR-499*** | ***miR-196a2*** | ***miR-146a*** | ***miR-149*** |  |  |  |  |  |
| 1  2  3  4  5  6  7  8  9  10  11  12  13  14  15 | T  T  T  T  T  T  T  T  C  C  C  C  C  C  C | C  C  T  C  T  T  T  C  C  T  C  T  C  C  T | G  C  G  G  G  C  C  C  C  G  G  C  G  C  G | T  T  T  C  C  T  C  C  C  T  T  T  C  T  C | 0.1591  0.1184  0.1501  0.0993  0.0862  0.0666  0.0582  0.0784  0.0255  0.0408  0.0138  0.0316  0.0284  0.0233  0.0201 | 0.1548  0.1453  0.0857  0.1333  0.0722  0.0959  0.0782  0.0499  0.0569  0.0181  0.0549  0.0158  0.0175  0.0131  0 | 0.1602  0.1299  0.1247  0.1109  0.0773  0.0761  0.0706  0.0699  0.0364  0.0292  0.029  0.0269  0.0256  0.0187  0.0144 | 1.00  1.09 (0.48 - 2.50) 0.40 (0.15 - 1.05)  0.96 (0.38 - 2.45)  0.70 (0.34 - 1.42)  1.08 (0.51 - 2.25)  1.35 (0.56 - 3.24)  0.47 (0.15 - 1.47)  2.50 (0.60 - 10.49)  0.44 (0.11 - 1.80)  3.22 (0.31 - 33.92)  0.46 (0.12 - 1.81)  0.42 (0.06 - 3.09)  0.34 (0.03 - 3.61)  0.00 (-Inf - Inf) | ---  0.84  0.063  0.94  0.32  0.85  0.5  0.2  0.21  0.25  0.33  0.27  0.39  0.37  1 |
| Global haplotype association *P*-value: 0.014 | | | | | | | | | |

Freq: frequency of haplotype, OR: odds ratios, N: number of subjects, CI: confidence intervals.

**Table S5.** Distribution of the *miR-146a* C > G, *miR-149* T > C, *miR-196a2* T > C and *miR-499* T > C SNPs allele and genotype frequencies in the pulmonary TB group and the control group in a Southern Han population

| **SNP sites** | **Allele** | **Controls N(Freq)** | **Patients N(Freq)** | ***P* value** | **OR (95% CI)** |
| --- | --- | --- | --- | --- | --- |
| *miR-146a* C > G Allele | C | 180(0.508) | 248(0.556) | 0.180 | 1  0.83 (0.62-1.09) |
|  | G | 174(0.492) | 198(0.444) |  |  |
| Genotype | CC | 41(0.232) | 64(0.287) |  | 1 |
|  | GC | 98(0.554) | 120(0.538) | 0.315 | 0.78 (0.49-1.26) |
|  | GG | 38(0.215) | 39(0.175) | 0.166 | 0.66(0.36-1.19) |
|  | HWE(*P*) | 0.152 | 0.179 |  |  |
| *miR-149* T > C Allele | T | 385(0.642) | 441(0.623) | 0.483 | 1  1.084(0.86-1.36) |
|  | C | 215(0.358) | 267(0.377) |  |  |
| Genotype | TT | 122(0.407) | 137(0.387) |  | 1 |
|  | TC | 141(0.470) | 167(0.472) | 0.753 | 1.06(0.76-1.47) |
|  | CC | 37(0.123) | 50(0.141) | 0.459 | 1.20(0.74-1.97) |
|  | HWE(*P*) | 0.703 | 0.938 |  |  |
| *miR-196a2* T > C Allele | T | 250(0.525) | 291(0.584) | 0.063 | 1  0.79(0.61-1.01) |
|  | C | 226(0.475) | 207(0.416) |  |  |
| Genotype | TT | 61(0.256) | 79(0.317) |  | 1 |
|  | TC | 128(0.538) | 133(0.534) | 0.296 | 0.80 (0.53-1.21) |
|  | CC | 49(0.206) | 37(0.149) | 0.050 | 0.58 (0.34-1.00) |
|  | HWE(*P*) | 0.227 | 0.116 |  |  |
| *miR-499* T > C Allele | T | 386(0.808) | 421(0.845) | 0.118 | 1  0.77 (0.55-1.07) |
|  | C | 92(0.192) | 77(0.155) |  |  |
| Genotype | TT | 156(0.614) | 177(0.711) |  | 1 |
|  | TC | 74(0.310) | 67(0.269) | 0.262 | 0.80(0.54-1.18) |
|  | CC | 9(0.035) | 5(0.020) | 0.201 | 0.49(0.16-1.49) |
|  | HWE(*P*) | 0.392 | 0.644 |  |  |

SNP: single nucleotide polymorphism, HWE: Hardy-Weinberg equilibrium, OR: odds ratios, CI: confidence intervals, N: number of alleles, Freq: frequency. All of the miRNA SNPs were in Hardy-Weinberg equilibrium.

**Table S6.** Association of *miR-146a* C > G, *miR-149* T > C, *miR-196a2* T > C and *miR-499* T > C SNPs with pulmonary TB in a Southern Han population using logistic regression

| **Model^a^** | **Genotype** | **Controls** | **Patients** | **OR (95% CI)** | ***P* value** | **AIC** | **BIC** |
| --- | --- | --- | --- | --- | --- | --- | --- |
| *miR-146a* C > G Codominant | C/C  G/C  G/G | 41(23.3%)  98 (55.7%)  37 (21%) | 64 (28.7%)  120 (53.8%)  39 (17.5%) | 1.00  0.78 (0.49-1.26)  0.68 (0.37-1.23) | 0.41 | 551.8 | 563.7 |
| Dominant | C/C  G/C-G/G | 41 (23.3%)  135(76.7%) | 64 (28.7%)  159 (71.3%) | 1.00  0.75 (0.48-1.19) | 0.22 | 550.1 | 558.1 |
| Recessive | C/C  G/G -G/C | 139 (79%)  37 (21%) | 184 (82.5%)  39 (17.5%) | 1.00  0.80 (0.48-1.31) | 0.37 | 550.8 | 558.8 |
| Overdominant | C/C-G/G  G/C | 78 (44.3%)  98 (55.7%) | 103 (46.2%)  120 (53.8%) | 1.00  0.93 (0.62-1.38) | 0.71 | 551.4 | 559.4 |
| Log-additive | --- | --- | --- | 0.82 (0.61-1.10) | 0.19 | 549.8 | 557.8 |
| *miR-149* T > C Codominant | T/T  T/C  C/C | 122(40.8%)  140(46.8%)  37 (12.4%) | 137 (38.7%)  167 (47.2%)  50 (14.1%) | 1.00  1.06 (0.76-1.48)  1.20 (0.74-1.96) | 0.76 | 906.1 | 919.5 |
| Dominant | T/T  T/C-C/C | 122(40.8%)  177(59.2%) | 137 (38.7%)  217 (61.3%) | 1.00  1.09 (0.80-1.50) | 0.58 | 904.3 | 913.3 |
| Recessive | T/T-T/C  C/C | 262(87.6%)  37 (12.4%) | 304 (85.9%)  50 (14.1%) | 1.00  1.16 (0.74-1.84) | 0.51 | 904.2 | 913.1 |
| Overdominant | T/T-C/C  T/C | 159(53.2%)  140(46.8%) | 187 (52.8%)  167 (47.2%) | 1.00  1.01 (0.75-1.38) | 0.93 | 904.6 | 913.6 |
| Log-additive | --- | --- | --- | 1.09 (0.87-1.36) | 0.47 | 904.1 | 913.1 |
| *miR-196a2* T > C Codominant | T/T  T/C  C/C | 61 (25.7%)  128 (54%)  48 (20.2%) | 79 (31.7%)  133 (53.4%)  37 (14.9%) | 1.00  0.80 (0.53-1.21)  0.60 (0.35-1.03) | 0.17 | 675.9 | 688.5 |
| Dominant | T/T  T/C-C/C | 61 (25.7%)  176(74.3%) | 79 (31.7%)  170 (68.3%) | 1.00  0.75 (0.50-1.11) | 0.14 | 675.3 | 683.7 |
| Recessive | T/T-T/C  C/C | 189(79.8%)  48 (20.2%) | 212 (85.1%)  37 (14.9%) | 1.00  0.69 (0.43-1.10) | 0.12 | 675 | 683.4 |
| Overdominant | T/T-C/C  T/C | 109 (46%)  128 (54%) | 116 (46.6%)  133 (53.4%) | 1.00  0.98 (0.68-1.39) | 0.9 | 677.4 | 685.8 |
| Log-additive | --- | --- | --- | 0.78 (0.59-1.01) | 0.061 | 673.9 | 682.3 |
| *miR-499* T > C Codominant | T/T  T/C  C/C | 156(61.4%)  74 (31%)  9 (3.5%) | 177 (71.4%)  66 (26.6%)  5 (2%) | 1.00  0.79 (0.53-1.17)  0.49 (0.16-1.49) | 0.25 | 678.2 | 690.7 |
| Dominant | T/T  T/C-C/C | 156(61.4%)83 (34.7%) | 177 (71.4%)  71 (28.6%) | 1.00  0.75 (0.51-1.11) | 0.15 | 676.9 | 685.2 |
| Recessive | T/T-T/C  C/C | 230(96.2%)  9 (3.5%) | 243 (98%)  5 (2%) | 1.00  0.53 (0.17-1.59) | 0.25 | 677.6 | 686 |
| Overdominant | T/T-C/C  T/C | 165 (65%)  74 (35%) | 182 (73.4%)  66 (26.6%) | 1.00  0.81 (0.55-1.20) | 0.29 | 677.8 | 686.2 |
| Log-additive | --- | --- | --- | 0.76 (0.54-1.06) | 0.1 | 676.3 | 684.7 |

^a^Adjusted for age and sex. OR: odds ratios, CI: confidence intervals, AIC: Akaike’s Information Criterion, BIC: Bayesian Information Criterion.
